# Vascular Access Perception and Quality of Life of Haemodialysis Patients

**DOI:** 10.3390/jcm13082425

**Published:** 2024-04-21

**Authors:** Kamil Sikora, Agnieszka Zwolak, Robert Jan Łuczyk, Agnieszka Wawryniuk, Marta Łuczyk

**Affiliations:** 1 Department of Internal Medicine and Internal Nursing, Chair of Preventive Nursing, Faculty of Health Sciences, Medical University of Lublin, Ul. Chodźki 7, 20-093 Lublin, Poland; 2 Department of Long-Term Care Nursing, Chair of Preventive Nursing, Faculty of Health Sciences, Medical University of Lublin, Ul. Chodźki 7, 20-093 Lublin, Poland

**Keywords:** quality of life, renal dialysis, vascular fistula, central venous catheter, functional status

## Abstract

**Background**: Patient quality of life is widely used as a non-clinical determinant of care. For patients undergoing hemodialysis, vascular access is vital to the delivery of hemodialysis and its function may affect not only the clinical outcome of treatment but also the overall quality of life of the patient, highlighting the need for increased efforts to improve the quality of hemodialysis vascular access care. The objective of this study was to evaluate the correlation between vascular access perception and quality of life in patients undergoing hemodialysis. **Methods**: A total of 202 patients with active hemodialysis vascular access were included in the study. Quality of life was assessed using the Kidney Disease Quality of Life Instrument (KDQOL™) questionnaire, while vascular access perception was evaluated using the Vascular Access Questionnaire (VAQ). **Results**: The study presented evidence on the influence of vascular access for hemodialysis patients on their quality of life. This impact is related to factors directly associated with vascular access, such as the type of access and the patient’s subjective evaluation of the access. **Conclusions**: The perception of vascular access is one of the factors that determines the quality of life of hemodialysis patients. The quality of life of hemodialysis patients decreases as the number of vascular access-related problems increases.

## 1. Introduction

Patient opinions on the impact of the healthcare they receive are increasingly being used as a measure of the effectiveness of clinical decision-making. Haemodialysis patients understand the significance of vascular access (VA) to renal replacement therapy and its impact on health-related quality of life (HRQOL). Although commonly used tools for assessing the quality of life of dialysis patients, such as the Kidney Disease Quality of Life Instrument (KDQOL™), include elements related to vascular access, its impact on HRQOL has not been sufficiently assessed using current tools. This highlights the need for further research in this area [1,2,3,4]. Examples of such assessments include patient-reported outcome measures (PROMs) and patient-reported experience measures (PREMs). These methods are standardized for assessing the impact of vascular access on a patient’s health status and, consequently, their health-related quality of life [3].

Due to the complexity of hemodialysis, vascular access can have a significant impact on the quality of life of patients. One modifiable factor that influences the HRQOL of patients undergoing dialysis is the type of vascular access used. Studies in this area have shown that the type of vascular access can affect patients’ quality of life, although the results are not yet conclusive. The impact on quality of life extends beyond the type of vascular access used, and includes physical symptoms such as pain, bleeding, bruising, and oedema, as well as complications like thrombosis and infection. Patient satisfaction with access and social functioning are also important factors to consider [5,6,7]. The study took all of these factors into consideration. The objective of this study was to evaluate the correlation between vascular access perception and quality of life in patients treated by hemodialysis.

## 2. Materials and Methods

The study was conducted from January 2021 to December 2022. The study group consisted of 120 respondents undergoing renal replacement therapy by hemodialysis at a dialysis center in Lublin, Poland, and 112 respondents from other centers in Poland who were part of an online community of dialysis patients. In the main stage of the study, 232 people were included in the total size of the study group. Out of these, 202 respondents’ answers qualified for statistical analysis.

The inclusion criteria were informed consent to participate in the study, age above 18 years, chronic kidney disease treated by hemodialysis, and having active vascular access for hemodialysis. The study protocol was approved by the Bioethics Committee of the Medical University of Lublin, Resolution No. KE-0254/178/2021 of 24 June 2021.

For the study, we used various diagnostic survey methods, including an auditorium survey technique, a distributed survey, an online survey, and a face-to-face interview technique. The research tools used in the study included a self-administered survey questionnaire that contained questions on sociodemographic variables such as age, gender, and type of vascular access.

The Vascular Access Questionnaire (VAQ) is a tool used to assess objective and subjective factors related to the functioning of vascular access for dialysis. It contains 17 potential problems perceived by the patient, each scored on a 5-point Likert scale. A score of 1 indicates that the vascular access problem has not bothered the patient in the past 4 weeks, while a score of 5 indicates that it has bothered the patient enormously. The summed score helps to determine the severity of vascular access function problems [8,9].

The Kidney Disease Quality of Life Instrument—Short Form (KDQOL-SF) is a standardized survey used to evaluate the quality of life of patients with kidney disease. The questionnaire comprises 24 questions, categorized by general perceptions of one’s health, information about kidney disease, its impact on daily life, and satisfaction with care. The initial section of the questionnaire utilizes the SF-36 quality of life assessment tool, which comprises 36 statements divided into 11 categories. The second section of the KDQOL-SF questionnaire evaluates the effect of kidney disease on the participant’s life and their contentment with the care they receive [10,11].

Statistical analyses were conducted using the R programming language version 4.2.2 and the RStudio environment version 2022.12.0. A significance level of α < 0.05 was used. The results for vascular access problems (VAQ), quality of life of the subjects (KDQOL-SF), duration of disease, dialysis, and age of the subjects were described using mean, standard deviation, median, mean rank, skewness, and kurtosis coefficients. The results for demographic variables and medical data of the subjects were statistically described using count and percentage distributions. To assess the relationship between the subjects’ quality of life and variables characterizing vascular access, Kruskal–Wallis tests were employed. Pearson’s r correlation coefficient and Gamma correlation coefficient were used to verify the associations between vascular access problems and quality of life of the domains.

## 3. Results

The study included 202 hemodialysis patients, of whom 51.98% (105 patients) were female and 48.02% (97 patients) were male. The mean age of the patients was 52.78 years (SD = 16.52), with a median of 51 years. The youngest patient was 21 years old, and the oldest was 92 years old. The mean duration of dialysis was 5.87 years (SD = 8.75), with a median of 2.67 years. The duration of dialysis ranged from less than one month to over 53 years. The age of patients and duration of dialysis are presented in Table 1 below.

The majority of patients had an arteriovenous fistula created from their own vessels (AVF), while a tunneled central venous catheter (CVC) was also common. Table 2 displays the characteristics of the different types of vascular access that patients had.

The study assessed vascular access problems using the VAQ questionnaire among hemodialysis patients. The mean severity of vascular access problems in the study group was 13.79, with a standard deviation of 11.22 and a median of 11. The study group comprised patients with different levels of severity of vascular access problems, from lack of problems to highly impactful problems (range 0–62). The study group of hemodialysis patients showed statistically significant differences in the severity of vascular access problems compared to a normal distribution. The severity of vascular access problems reported by respondents was moderately low. Table 3 presents the aforementioned data.

The quality of life of the patients was assessed using the KDQOL-SF questionnaire, and the relationhips between the quality of life of the patients and the severity of vascular access problems were investigated. The higher the score on the KDQOL-SF questionnaire scales, the better the quality of life in a specific domain. The scale names were slightly modified to better reflect this feature.

Table 4 presents the participants’ quality of life scores, as measured by the first part of the KDQOL-SF questionnaire.

The scores resulting from the distributions significantly deviated from the normal distribution, with significant deviations occurring in the scales of freedom in performing roles for emotional reasons, for health reasons, and for physical functioning.

Table 5 displays the subjects’ quality of life distribution in the area related to kidney problems (the second part of the KDQOL-SF questionnaire).

The scores on the analyzed scales resulted in distributions that were significantly different from the normal distribution, with significant deviations observed in work status.

Table 6 displays the correlation between the patients’ overall quality of life and the type of hemodialysis vascular access.

The statistical analysis revealed small but significant correlations between the type of vascular access and the absence of pain. It is important to note that these findings are objective and do not reflect any subjective evaluations. The highest incidence of pain-free patients was observed in those with an arteriovenous fistula. Conversely, the lowest incidence was observed in those with a non-tunneled central venous catheter. Patients with a tunneled central venous catheter or an arteriovenous graft had intermediate levels of pain.

The study showed the following relationship between overall health and type of vascular access: those with an autologous arteriovenous fistula had the highest quality of life in the area of overall health, those with a non-tunneled central venous catheter had the lowest; those with a central venous catheter—tunneled—had the lowest; and those with a vascular prosthesis had the lowest. The statistical analysis revealed that the observed differences were both significant and small.

In terms of the relationship between vitality and type of vascular access, patients with an autologous arteriovenous fistula had the highest vitality, followed by patients with a vascular graft, lower vitality in patients with a non-tunneled central venous catheter, and the lowest vitality in patients with a tunneled central venous catheter. It is important to note that these findings are objective and not influenced by personal opinions or biases.

Regarding the relationship between social functioning and type of vascular access, the best social functioning was observed in patients with a vascular graft, followed by those with an arteriovenous fistula, then those with a non-tunneled central venous catheter, and the least by those with a tunneled central venous catheter. The Kruskal–Wallis test revealed statistically significant but small differences.

Table 7 presents the association between respondents quality of life and renal function problems, with consideration given to the type of vascular access.

The study analyzed the relationship between the quality of life of hemodialysis patients with kidney disease-related problems and the type of vascular access used. The results showed that patients with an arteriovenous fistula had the highest quality of life in relation to the effects of the disease, followed by those with a vascular prosthesis, those with a non-tunneled central venous catheter, and those with a tunneled central venous catheter, who had the lowest quality of life. The Kruskal–Wallis test indicated statistically significant and small differences.

Among hemodialysis patients, the type of vascular access is related to the quality of life related to disease burden. Subjects with an arteriovenous fistula from their own vessels had the highest level of quality of life in this domain, followed by those with a non-tunneled central venous catheter, then with a vascular graft, and finally those with a tunneled central venous catheter. The Kruskal–Wallis test indicated statistically significant but small differences.

Table 8 presents the association between the severity of vascular access problems and patients overall quality of life.

The severity of vascular access problems was associated relatively strongly with the absence of pain (r = −0.411; *p* = 0.000), moderately with emotional well-being (r = −0.399; *p* = 0.000), and social functioning (r = −0.392; *p* = 0). The results indicate a negative correlation between the participant’s general perception of health (r = −0.392; *p* = 0.000), social functioning (r = −0.392; *p* = 0.000), vitality (r = −0.370; *p* = 0.000), role freedom due to physical health (r = −0.283; *p* = 0.000), role freedom due to emotional reasons (γ = −0.272; *p* = 0.000), and a rather small correlation with physical functioning (r = −0.139; *p* = 0.049). The severity of vascular access problems negatively impacts various aspects of quality of life, including pain, mental health, social functioning, overall health perception, vitality, and the ability to perform physical and emotional roles.

Table 9 shows the association between the severity of vascular access problems and the patients’ quality of life related to kidney problems.

Correlation analyses revealed a significant association between the severity of vascular access problems and quality of life in the domains of disease effects (r = −0.515; *p* = 0.000), symptoms (r = −0.468; *p* = 0.000), and cognitive functioning (r = −0.432; *p* = 0.000), as well as moderate satisfaction with dialysis station staff care (r = −0.323; *p* = 0.000). The study found statistically significant negative correlations between the severity of vascular access problems and quality of sleep (r = −0.353; *p* = 0.000), quality of social relationships (r = −0.343; *p* = 0.000), quality of life in the area of disease burden (r = −0.333; *p* = 0.000), level of dialysis staff encouragement (r = −0.323; *p* = 0.000), and level of social support (r = −0.230; *p* = 0.001). However, there was no statistically significant relationship between the severity of vascular access problems and quality of life in the domain of work activity (γ = −0.098; *p* = 0.167). The severity of vascular access problems negatively impacts various aspects of a patient’s quality of life, including disease effects, symptoms, cognitive functioning, satisfaction with dialysis station staff care, sleep, social relationships, disease burden, patient support from dialysis station staff, and social support.

## 4. Discussion

Quality of life (QOL) and health-related quality of life (HRQOL) are recognized as important indicators of healthcare outcomes and determinants of biopsychosocial well-being. In hemodialysis patients, QOL is a predictor of disease progression, a valuable research tool in assessing care effectiveness, and a prognostic factor. Systematic measurements of quality of life and the variables that affect it can be helpful criteria in planning patient care [12,13,14].

The KDQOL-SF questionnaire is a commonly used tool to measure the quality of life of patients with end stage renal disease (ESRD). Our research used this tool to obtain results on quality of life in the study group. In the initial section of the questionnaire, which evaluates the overall quality of life of the participants, the patients reported the lowest quality of life in terms of physical functioning and the resulting limitations in their daily activities, as well as a general perception of health. The Dialysis Outcome and Practice Pattern Study (DOPPS) is a multicenter, international study based in the United States. The study found that low scores on the physical component of health-related quality of life (HRQOL) were associated with increased mortality and a higher risk of future hospitalization. Therefore, it is important to prioritize educating dialysis patients on the physical aspects of the disease [4,15,16].

In the part of the questionnaire assessing disease-related quality of life, respondents had the lowest QoL scores in terms of the perceived burden of chronic kidney disease and occupational activity. The above results are confirmed by the work of other researchers, who have also shown that one of the methods of improving patients’ occupational functioning may be a change in the type of renal replacement therapy [15,17].

The impact of vascular access functioning on the quality of life of hemodialysis patients was a relevant element in the study. This includes the type of vascular access currently in place, the number of previous accesses, problems with its use, the need for hospitalization, and the level of pain during direct care. The importance of vascular access to the quality of life of hemodialysis patients is evident from the patients’ perspective alone. The Standardized Outcomes in Nephrology-Hemodialysis (SONG-HD) study aimed to determine endpoints for the overall nephrology care of hemodialysis patients. One of the key assessment indicators affecting patients’ quality of life and function during dialysis was identified as vascular access. However, there are few widely used, specific tools to assess the impact of vascular access. In 2021, Richarz et al. developed the Vascular Access Specific Quality of Life Measure (VASQoL), while Nordyke et al. created the Hemodialysis Access-Related Quality of Life (HARQ) assessment tool. These tools may be useful in future studies, including our own [18,19,20,21,22,23].

Our study demonstrated the impact of vascular access perception on patients quality of life. The VAQ and KDQOL-SF questionnaire evaluations revealed that patients with more vascular access-related issues experienced a lower quality of life. The relationship between vascular access care provided by nurses and the overall well-being of the patient was noted in every domain of quality of life outside of work activity. This was particularly evident in the domain of symptoms and complications of the disease, physical and social aspects, and satisfaction with the overall care of dialysis station staff. These correlations highlight the significance of vascular access care in promoting patient well-being. Improving the quality of nursing care, with special attention paid to hemodialysis vascular access care, can help eliminate or reduce the incidence of problems associated with vascular access. However, factors beyond the control of the personnel caring for the patient, such as the type of vascular access, can also affect the patient’s quality of life and overall well-being. Our study, along with the work of other authors, has shown that the type of vascular access has an impact on the quality of life of hemodialysis patients. It is important to note that this is a subjective evaluation. Therefore, it is necessary to consider other factors when making a decision about the type of vascular access to use. Patients who preferred AVF as their type of vascular access had the highest overall perception of health and vitality. In 2019, Do Hyoung Kim et al. published a paper as part of a prospective cohort study conducted by the Clinical Research Center for End Stage Renal Disease (CRC for ESRD) in Korea. The study, which involved 1461 hemodialyzed patients across multiple centers, aimed to confirm previous findings. Do Hyoung Kim et al. found that patients with vascular access in the form of an arteriovenous fistula from their own vessels or a vascular prosthesis had higher quality of life scores than patients with CVC in 10 out of 12 quality of life domains after 3 months. After 12 months of dialysis therapy, there was an improvement in HRQOL score, highlighting the importance of regular assessment of HRQOL and its association with vascular access. A study conducted by Natalie Domenick Sridharan et al. on a group of 77 hemodialyzed patients found no effect of the type of vascular access on quality of life. The relationships observed were similar in patients with arteriovenous fistulas (AVFs), arteriovenous grafts (AVGs), and central venous catheters (CVCs). In contrast, patients with arteriovenous fistulas (AVFs) reported the highest satisfaction with their access, as measured by the VAQ questionnaire. Our study also found a correlation between vascular access perception and health-related quality of life. The duration of dialysis therapy and the past history of patients’ vascular access could also affect HRQOL. Patients who had previously undergone vascular access procedures, had been hospitalized due to complications related to vascular access, or reported recent issues with its functioning exhibited lower quality of life scores across all domains, including disease-related and overall. M. Pole et al. also recognized the aforementioned correlations. In their evaluation of 749 hemodialysis patients in the UK, they found a lower satisfaction rating for vascular access, as well as an increased number of complications, hospitalizations, or the need for intervention. Efforts to educate and implement vascular access care patterns should be intensified, particularly in nursing practice, to ensure that vascular access is not only effective but also contributes to the quality of life of renal replacement therapy patients. Studies have shown that choosing AVF as the primary access results in the lowest number of complications and the greatest impact on the overall well-being of the patient [2,7,9,24,25,26,27,28,29].

It is important to note the limitations of this study. The study group was limited to patients treated at dialysis centers in one country, which may limit the generalizability of the results. A minority of patients had vascular access using AVG and non-tunneled CVCs, compared to patients with AVF and tunneled CVCs. Additionally, the analysis did not consider objective factors related to vascular access function, such as arteriovenous fistula blood flow, laboratory results, or the adequacy of dialysis with access as measured by the Kt/V ratio. Consider adding the clinical variables mentioned above when conducting further studies in this subject area.

## 5. Conclusions

This impact is determined by both objective factors, such as the type of access, and subjective factors, such as the patient’s assessment of the access. The study demonstrated the impact of vascular access on the quality of life of hemodialysis patients. The perception of vascular access is a crucial element in determining the quality of life of hemodialysis patients. The quality of life of hemodialysis patients decreases as the number of vascular access-related problems increases.

## Figures and Tables

**Table 1:** Patient’s age and duration of dialysis (in years).

Variable	*n*	M	SD	Me	Min	Max	Skew	Kurt
Age	202	52.78	16.52	51	21	92	0.096	−0.975
Duration of dialysis	193	5.87	8.75	2.67	0.08	53.81	2.853	9.203

*n*—number of observations; M—mean; Me—median; SD—standard deviation; Skew—skewness coefficient; Kurt—kurtosis coefficient.

**Table 2:** Type of vascular access.

Type of Vascular Access	*n*	%
Arteriovenous Fistula (AVF)	134	66.34
Tunneled Central Venous Catheter (CVC)	58	28.71
Non-Tunneled Central Venous Catheter (CVC)	5	2.48
Arteriovenous Graft (AVG)	5	2.48

*n*—number of observations; %—percentage.

**Table 3:** Severity of vascular access problems.

Variable	*n*	M	SD	Me	Min	Max	Skew	Kurt
Severity of vascular access problems	202	13.79	11.22	11	0	62	1.289	1.755

*n*—number of observations; M—mean; Me—median; SD—standard deviation; Skew—skewness coefficient; Kurt—kurtosis coefficient.

**Table 4:** Overall quality of life of the respondents.

Quality of Life Domain	*n*	M	SD	Me	Min	Max	Skew	Kurt
Physical functioning	202	53.04	29.25	55.00	0	100	−0.312	−1.098
Freedom to perform roles due to physical health	202	32.05	39.70	0.00	0	100	0.788	−1.035
Absence of pain	202	61.74	28.44	59.50	0	100	−0.279	−0.948
General health perceptions	202	38.32	19.64	40.00	0	97	0.286	−0.215
Vitality	202	45.83	20.66	48.33	0	90	−0.113	−0.350
Social function	202	55.63	27.15	50.00	0	100	−0.236	−0.701
Freedom to perform roles for emotional reasons	202	49.34	45.62	33.33	0	100	0.035	−1.831
Emotional well-being	202	54.21	20.90	53.50	0	100	−0.239	−0.285

*n*—number of observations; M—mean; Me—median; SD—standard deviation; Skew—skewness coefficient; Kurt—kurtosis coefficient.

**Table 5:** Respondents Quality of Life in kidney disease targeted scales.

Scale	*n*	M	SD	Me	Min	Max	Skew	Kurt
Symptom/problems	202	69.88	17.34	70.83	6.25	100	−0.612	0.076
Effects of kidney disease	202	49.92	24.42	50.00	0.00	100	−0.123	−0.990
Burden of kidney disease	202	33.26	25.22	25.00	0.00	100	0.558	−0.563
Work status	202	31.68	40.96	0.00	0.00	100	0.758	−1.097
Cognitive function	202	63.96	24.52	66.67	0.00	100	−0.545	−0.206
Quality of social interaction	202	60.74	22.20	60.00	0.00	100	−0.475	−0.063
Sleep	202	49.20	20.31	50.00	5.00	100	0.079	−0.591
Social support	202	66.50	24.60	66.66	0.00	100	−0.592	−0.258
Dialysis staff encouragement	202	74.07	24.37	75.00	0.00	100	−0.822	0.201
Patient satisfaction	202	60.23	23.15	66.67	0.00	100	−0.368	−0.050

*n*—number of observations; M—mean; Me—median; SD—standard deviation; Skew—skewness coefficient; Kurt—kurtosis coefficient.

**Table 6:** Relationship between the overall quality of life of respondents and type of vascular access.

Quality of Life Domain	Type of Vascular Access	*n*	M	SD	Me	Mr	Kruskal–Wallis H Test			
							H	df	*p*	η^2^
Physical functioning	Non-Tunneled Central Venous Catheter	5	26.00	27.70	25.00	50.50	6.335	3	0.096	0.017
	Tunneled Central Venous Catheter	58	48.79	28.83	50.00	93.22				
	Arteriovenous Graft	5	60.00	36.23	60.00	118.50				
	Arteriovenous Fistula	134	55.63	28.81	60.00	106.35				
Freedom to perform roles due to physical health	Non-Tunneled Central Venous Catheter	5	20.00	44.72	0.00	78.20	3.586	3	0.310	0.003
	Tunneled Central Venous Catheter	58	25.00	35.36	0.00	92.87				
	Arteriovenous Graft	5	45.00	51.23	25.00	117.70				
	Arteriovenous Fistula	134	35.07	40.80	25.00	105.50				
Absence of pain	Non-Tunneled Central Venous Catheter	5	26.10	17.27	24.50	31.40	8.803	3	0.032	0.029
	Tunneled Central Venous Catheter	58	59.01	28.23	58.25	96.95				
	Arteriovenous Graft	5	58.40	23.57	47.00	90.00				
	Arteriovenous Fistula	134	64.38	28.23	69.00	106.51				
General health perceptions	Non-Tunneled Central Venous Catheter	5	37.40	15.61	35.00	97.40	9.687	3	0.021	0.034
	Tunneled Central Venous Catheter	58	32.61	17.07	35.00	84.53				
	Arteriovenous Graft	5	28.80	24.85	22.00	67.20				
	Arteriovenous Fistula	134	41.18	20.15	42.00	110.28				
Vitality	Non-Tunneled Central Venous Catheter	5	40.00	16.96	40.00	84.10	12.216	3	0.007	0.046
	Tunneled Central Venous Catheter	58	38.25	20.22	40.00	79.91				
	Arteriovenous Graft	5	49.00	13.87	50.00	110.80				
	Arteriovenous Fistula	134	49.22	20.43	50.00	111.15				
Social function	Non-Tunneled Central Venous Catheter	5	52.50	34.69	50.00	95.90	8.611	3	0.035	0.028
	Tunneled Central Venous Catheter	58	46.12	29.91	50.00	82.98				
	Arteriovenous Graft	5	62.50	31.87	62.50	113.80				
	Arteriovenous Fistula	134	59.61	24.62	62.50	109.26				
Freedom to perform roles for emotional reasons	Non-Tunneled Central Venous Catheter	5	26.67	43.46	.33.33	76.00	3.188	3	0.364	0.001
	Tunneled Central Venous Catheter	58	45.40	45.33	33.33	97.23				
	Arteriovenous Graft	5	26.67	43.46	.66.67	76.00				
	Arteriovenous Fistula	134	52.74	45.76	66.67	105.25				
Emotional well-being	Non-Tunneled Central Venous Catheter	5	52.00	20.59	52.00	95.90	7.020	3	0.071	0.020
	Tunneled Central Venous Catheter	58	47.64	20.61	52.00	84.69				
	Arteriovenous Graft	5	55.20	22.16	56.00	104.20				
	Arteriovenous Fistula	134	57.10	20.57	56.00	108.88				

*n*—number of observations; M—mean; SD—standard deviation; Me—median; Mr—mean rank; H—result of the Kruskal–Wallis H-test; df—degrees of freedom; *p*—test probability; η^2^—effect size; eta—square.

**Table 7:** The association between the quality of life of the respondents and problems related to kidney function and the type of their vascular access.

Scale	Group	*n*	M	SD	Me	Mr	Kruskal–Wallis H Test			
							H	df	*p*	η^2^
Symptom/problems	Non-Tunneled Central Venous Catheter	5	60.83	16.50	56.25	67.50	2.418	3	0.490	0.003
	Tunneled Central Venous Catheter	58	69.03	18.39	70.83	98.69				
	Arteriovenous Graft	5	66.67	17.74	66.67	88.60				
	Arteriovenous Fistula	134	70.71	16.95	72.92	104.47				
Effects of kidney disease	Non-Tunneled Central Venous Catheter	5	46.25	21.23	57.14	91.30	10.268	3	0.016	0.037
	Tunneled Central Venous Catheter	58	41.29	24.10	40.62	81.71				
	Arteriovenous Graft	5	47.86	20.22	40.62	93.40				
	Arteriovenous Fistula	134	53.86	24.04	56.25	110.75				
Burden of kidney disease	Non-Tunneled Central Venous Catheter	5	32.50	24.37	25.00	103.60	9.177	3	0.027	0.031
	Tunneled Central Venous Catheter	58	25.43	23.50	18.75	82.67				
	Arteriovenous Graft	5	30.00	33.48	18.75	89.20				
	Arteriovenous Fistula	134	36.80	25.17	31.25	110.03				
Work status	Non-Tunneled Central Venous Catheter	5	20.00	27.39	0.00	91.10	0.833	3	0.841	0.011
	Tunneled Central Venous Catheter	58	31.90	40.50	0.00	102.24				
	Arteriovenous Graft	5	20.00	44.72	0.00	83.70				
	Arteriovenous Fistula	134	32.46	41.70	0.00	102.23				
Cognitive function	Non-Tunneled Central Venous Catheter	5	56.00	28.52	73.33	86.90	1.622	3	0.655	0.007
	Tunneled Central Venous Catheter	58	60.23	27.53	63.33	95.32				
	Arteriovenous Graft	5	60.00	27.08	60.00	91.60				
	Arteriovenous Fistula	134	66.02	22.89	66.67	105.09				
Quality of social interaction	Non-Tunneled Central Venous Catheter	5	65.33	15.92	73.33	114.70	5.552	3	0.136	0.013
	Tunneled Central Venous Catheter	58	54.60	24.83	60.00	87.43				
	Arteriovenous Graft	5	53.33	23.57	60.00	85.40				
	Arteriovenous Fistula	134	63.51	20.72	66.67	107.70				
Sleep	Non-Tunneled Central Venous Catheter	5	46.00	15.37	37.50	91.30	4.574	3	0.206	0.008
	Tunneled Central Venous Catheter	58	44.76	21.61	40.00	88.38				
	Arteriovenous Graft	5	55.50	26.60	65.00	115.60				
	Arteriovenous Fistula	134	51.00	19.53	51.25	107.03				
Social support	Non-Tunneled Central Venous Catheter	5	73.33	9.13	66.66	110.20	4.273	3	0.233	0.006
	Tunneled Central Venous Catheter	58	63.22	25.89	66.66	96.81				
	Arteriovenous Graft	5	49.99	20.41	49.99	54.90				
	Arteriovenous Fistula	134	68.28	24.34	66.66	104.94				
Dialysis staff encouragement	Non-Tunneled Central Venous Catheter	5	57.50	32.60	50.00	68.60	4.921	3	0.178	0.010
	Tunneled Central Venous Catheter	58	73.71	24.30	75.00	100.21				
	Arteriovenous Graft	5	60.00	13.69	50.00	59.10				
	Arteriovenous Fistula	134	75.37	24.23	75.00	104.87				
Patient satisfaction	Non-Tunneled Central Venous Catheter	5	50.00	0.00	50.00	65.00	4.608	3	0.203	0.008
	Tunneled Central Venous Catheter	58	62.64	23.84	66.67	106.78				
	Arteriovenous Graft	5	50.00	0.00	50.00	65.00				
	Arteriovenous Fistula	134	59.95	23.58	66.67	101.94				

*n*—number of observations; M—mean; SD—standard deviation; Me—median; Mr—mean rank; H—result of the Kruskal–Wallis H-test; df—degrees of freedom; *p*—test probability; η^2^—effect size eta—square.

**Table 8:** Severity of vascular access problems and patients overall quality of life.

Quality of Life Domain	Severity of Vascular Access Problems	
	r/γ	*p*
Physical functioning	−0.139	0.049
Freedom to perform roles due to physical health	−0.283	0.000
Absence of pain	−0.411	0.000
General health perceptions	−0.382	0.000
Vitality	−0.370	0.000
Social function	−0.392	0.000
Freedom to perform roles for emotional reasons	−0.272	0.000
Emotional well-being	−0.399	0.000

r—Pearson’s correlation coefficient r; γ—Gamma correlation coefficient; *p*—test probability.

**Table 9:** Vascular access problem, severity, and patients’ kidney-related quality of life.

Scale	Severity of Vascular Access Problems	
	r/γ	*p*
Symptom/problems	−0.468	0.000
Effects of kidney disease	−0.515	0.000
Burden of kidney disease	−0.333	0.000
Work status	−0.098	0.167
Cognitive function	−0.432	0.000
Quality of social interaction	−0.343	0.000
Sleep	−0.353	0.000
Social support	−0.230	0.001
Dialysis staff encouragement	−0.323	0.000
Patient satisfaction	−0.358	0.000

r—Pearson’s correlation coefficient r; γ—Gamma correlation coefficient; *p*—test probability.

## Data Availability

Data are contained within the article.
